# Fallbasiertes Lernen kann die Lehrqualität im unfallchirurgischen Unterricht verbessern: Eine Umfrageanalyse unter Medizinstudierenden

**DOI:** 10.1007/s00113-021-01009-3

**Published:** 2021-06-08

**Authors:** Friedemann Strobel, Tina Histing, Tim Pohlemann, Antonius Pizanis, Benedikt Johannes Braun, Marcel Orth, Tobias Fritz

**Affiliations:** 1 Klinik für Unfall‑, Hand- und Wiederherstellungschirurgie, Universitätsklinikum des Saarlandes, Kirrberger Str. 1, 66421 Homburg/Saar, Deutschland; 2 Klinik für Anästhesiologie und operative Intensivmedizin, Kliniken Köln-Merheim, Ostmerheimer Str. 200, 51109 Köln, Deutschland

**Keywords:** Chirurgische Lehre, Fallbasiertes Lernen, Studierendenzentriertes Lehrformat, Seminar Unfallchirurgie, Lehre, Surgical teaching, Case-based learning, Student-centered teaching format, Trauma surgery seminar, Teaching

## Abstract

**Hintergrund:**

Medizinische Lehre ist seit jeher eine Herausforderung für Studierende und Dozenten. Die Förderung des Zusammenhangswissens und ein Wissenstransfer von der Theorie auf die Praxis gewinnen in den letzten Jahren an Bedeutung. Um dieses Ziel zu erreichen, werden zunehmend studierendenzentrierte Lehrkonzepte in der Literatur eingesetzt.

**Fragestellung:**

Kann durch ein fallbasiertes Lehrkonzept das unfallchirurgische Seminar verbessert werden?

**Material und Methoden:**

Den Studierenden und Dozenten wurden standardisierte Fallbeispiele und dazugehörige Unterrichtsmaterialien wie Klassifikationshilfen und Versorgungsstrategien zur Verfügung gestellt. Durch eine zweizeitige Evaluation konnten die Auswirkungen dieser Modifikationen des Lehrdesigns erfasst und statistisch ausgewertet werden.

**Ergebnisse:**

Das Seminar wurde als prüfungsrelevanter empfunden. Die Lehre durch die Dozenten wurde als kompetenter und motivierter empfunden. Insgesamt wurde das Seminar durch die Studierenden besser bewertet.

**Schlussfolgerungen:**

Ein fallbasiertes Lehrkonzept kann daher, richtig und gezielt eingesetzt, die unfallchirurgische Lehre signifikant verbessern.

## Abstract (en)

**Background:**

Medical education has always been challenging for students and teachers. Of growing importance is the promotion of knowledge of correlations and knowledge transfer from theoretical aspects into clinical practice. In order to achieve this aim, student-centered teaching concepts are increasingly being employed in the literature.

**Objective:**

Can a trauma surgery practical seminar be improved by a case-based teaching concept?

**Material and methods:**

For this purpose, standardized case studies and corresponding teaching materials, such as classification aids and treatment strategies, were made available to the students and lecturers. Using a two-staged evaluation the effects of the modified teaching design could be recorded and statistically analyzed.

**Results:**

The seminar was considered to be relevant for the examinations. The teaching by the lecturers was found to be more competent and appeared more motivated. Overall, the seminar was rated better by the students.

**Conclusion:**

A case-based teaching concept can significantly improve the education in trauma surgery, when correctly and specifically implemented.

## Einführung

Die Gestaltung der medizinischen Lehre ist seit jeher eine Herausforderung für Studierende und Lehrende. Ein Großteil der medizinischen Lehre erfolgt hierbei auch heute noch über eher dozentenzentrierte Lehrmodelle, z. B. durch Vorlesungen [13]. Allerdings steigt die Bedeutung in der medizinischen Lehre für studierendenzentrierte Lehrmethoden gegenüber den traditionellen dozentenzentrierten Modellen, da hierdurch der Lehrinhalt praxisnäher gestaltet werden kann [7]. Durch die Förderung des Zusammenhangswissens soll der Wissenstransfer von der Theorie in die Praxis besser gelingen [7, 21].

Eine Möglichkeit hierfür ist der Einsatz von fallbasiertem Lernen. Hierbei bearbeiten Studierende strukturierte Fallbeispiele. Der Lehrende nimmt in der anschließenden Diskussion die Rolle des Moderators ein, um diese entsprechend zu steuern [7]. Dadurch kann der Lehrende Einfluss auf den Fokus relevanter Aspekte nehmen und gewährleistet einen strukturierten und effizienten Ablauf der Falllösung [7].

Um die positiven Effekte des fallbasierten Lernens zu nutzen und dadurch eine weniger vom Dozenten abhängige Lehre zu erreichen, war es das Ziel dieser Arbeit, diese in das unfallchirurgische Curriculum zu integrieren.

Im Rahmen des unfallchirurgischen Praxisseminars des Studiengangs Humanmedizin soll praxisnahes Wissen vermittelt werden. Bis zur Umstellung auf das hier beschriebene fallbasierte Lernen war das unfallchirurgische Praxisseminar in 6 Unterrichtsthemen mit je 90 min 2‑mal/Woche im 1. bzw. 2. klinischen Semester etabliert. Abschließend erfolgte eine Gesamtklausur über die Inhalte aller chirurgischen Praxisseminare (Allgemein‑, Viszeral‑, Gefäß‑, Kinder‑, Neuro‑, Herz‑, Thorax- und Unfallchirurgie). Das Praxisseminar Unfallchirurgie konnte daher nicht eigenständig auf andere Prüfungsmodalitäten umgestellt werden (z. B. OSCE). Der Umstellung auf fallbasiertes Lernen war eine Analyse der eingesetzten Unterrichtsmaterialien der Dozenten des Praxisseminars vorangegangen. Hierbei zeigten sich strukturelle und inhaltliche Unterschiede. Bis zur Restrukturierung war es den Dozenten freigestellt, welchen Lehransatz und welche Lehrmittel sie verwenden wollten. Lediglich das vorgegebene Themengebiet sollte behandelt werden. Eine standardisierte Ausbildung der Studierenden konnte damit nicht in allen Fällen gewährleistet werden. Fallbasiertes Lernen und problemorientiertes Lernen (POL) wurden am Lehrstuhl als freiwilliges Lehrangebot angeboten. Die darin enthaltenen Inhalte wurden außerdem für eine internetbasierte Lernplattform im Sinne des distributiven problemorientierten Lernens (dPOL) verwendet [3, 11, 12].

Ziel des Projektes war es, für alle Seminarteilnehmer eine äquivalente, qualitativ hochwertige Lehre zu gewährleisten. Darüber hinaus sollten Dozenten mit unterschiedlicher Berufserfahrung eine Struktur zur Verfügung gestellt werden, die eine solche Lehre sicherstellt. Ein Ansatz, um dies zu erreichen, ist die Etablierung eines dozenten-/studierendenzentrierten balancierten Lehrkonzepts mit fallbasiertem Lernen.

## Material und Methoden

### Kasuistiken

**Figure 1:**
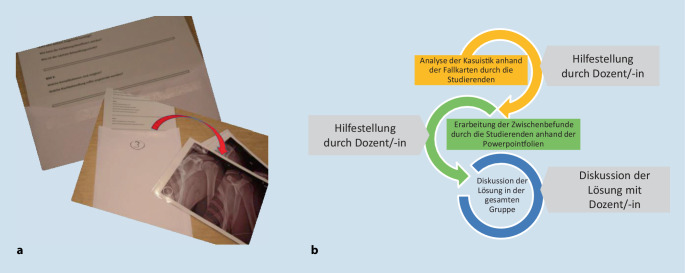

Anhand der DGUV-Unfallstatistik 2016 [26] wurden häufige und für den klinischen Alltag repräsentative Verletzungsarten ausgewählt. Diese wurden anschließend als repräsentative anonymisierte Fallbeispiele ausgearbeitet (Infobox 1). Zu den einzelnen Fallbeispielen wurden Bearbeitungsanleitungen und Klassifikationshilfen erstellt. Gegliedert war jedes Fallbeispiel in die Abschnitte Anamnese/Fallbeschreibung und Diagnostik (inklusive anonymisierter Röntgen‑, CT- und MRT-Bilder). Aus den Unterlagen wurden entsprechende Fallkarten im DIN-A4-Format ausgedruckt und den Studierenden für das Seminar zur Verfügung gestellt (Abb. 1). Außerdem wurde für jedes Fallbeispiel eine Kasuistik als Präsentation (PowerPoint; Fa. Microsoft, Redmond, WA, USA) erstellt.

### Seminarablauf

Der Unterricht umfasste 7 Termine mit je 90 min während des Wintersemesters 2017/2018. Hierbei waren 2 Termine für praktische Inhalte wie Nahtkurs und Untersuchungstechniken vorgesehen. Die weiteren 5 Termine waren als Seminar geplant. Die Dozenten (*n* = 20) waren klinisch tätige Ärztinnen und Ärzte der Unfallchirurgie vom 1. bis 28. Berufsjahr. Vor Durchführung des Semesters erfolgte eine 60-minütige Instruktion aller Dozenten über die neue Seminarstruktur. Das Praxisseminar war in Gruppen mit je 20 ± 2 Studierenden aufgeteilt. Innerhalb der Seminargruppe wurden, entsprechend der Literaturempfehlung, Kleingruppen zu je 4 bis 5 Personen gebildet [16, 23]. Jeder Gruppe wurde ein Fall zugewiesen. Innerhalb von 15 min sollten eine Verdachtsdiagnose gestellt und Vorschläge für eine fachgerechte Diagnostik, Klassifikation und Therapie entwickelt werden. Fragen an bzw. Hilfestellung durch die Dozenten waren jederzeit möglich. Nach erfolgreicher Bearbeitung der Fälle erfolgte durch die Studierenden eine Vorstellung der Kasuistik mithilfe der Fallpräsentationen.

### Evaluation/Auswertung

Ein standardisierter Evaluationsbogen der Universität für Seminare (Infobox 2) wurde bereits im Sommersemester 2016 verwendet, in welchem der Unterricht nach alten Vorgaben ohne einheitliche Kasuistik erfolgte und diente als Kontrollgruppe. Der gleiche Evaluationsbogen des Sommersemesters 2016 wurde mit dem des Wintersemesters 2017/2018 verglichen (Infobox 2). Darüber hinaus wurde für das Projekt ein zusätzlicher Evaluationsbogen erstellt und durch die Studierenden nach Abschluss des Semesters beantwortet (Infobox 3). Das Ziel war es, zunächst Kollektivdaten wie Alter und Geschlecht zu evaluieren (Infobox 3). Dann wurden die Berufswunsch-Präferenzen der Studierenden erfragt (Infobox 3). Abschließend erfolgten spezifische Fragen bezüglich des Seminarkonzeptes (Infobox 3).

Die Fragen wurden so konzipiert, dass sie mit einer klaren Ja- oder Nein-Antwort beantwortet werden konnten, um eine Ablehnung oder Befürwortung klar zu definieren.

### Statistik

Die statistische Auswertung erfolgte mittels Microsoft Excel(Fa. Microsoft, Redmond, WA, USA) und Sigma Plot 13 (Fa. Systat, San José, CA, USA). Die Analyse zwischen den Gruppen erfolgte mittels zweiseitigem *t*-Test; dieser wurde bei signifikantem Unterschied um einen einseitigen *t*-Test ergänzt. Das Signifikanzniveau wurde mit *p* < 0,05 festgelegt.

## Ergebnisse

**Figure 2:**
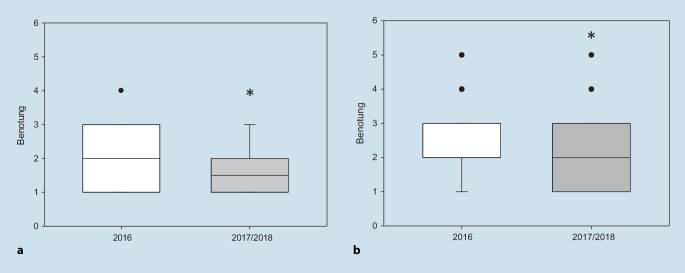

Insgesamt zeigte sich die Anzahl ausgewerteter Evaluationsbogen der Universität mit *n*_2017_ = 80 vs. *n*_2016_ = 116. Die standardisierten Fragen waren in beiden Jahrgängen gleich erstellt, lediglich die Reihenfolge auf dem Evaluationsbogen wich voneinander ab. Das Curriculum war in beiden Jahrgängen vor dem ersten Termin des Praxisseminars offiziell bereitgestellt, sodass die Studierenden dieses auch in beiden Jahren bestätigten. In der Auswertung der beiden unterschiedlichen Semester zeigten sich signifikant bessere Bewertungen des neu strukturierten Seminars für die Veranstaltung insgesamt, der Definition der Lernziele, der Prüfungsrelevanz, der Motivation und Vorbereitung des Dozenten, der Wiederholung der Inhalte, der Bereitschaft zur Diskussion und des Tempos des Dozenten (Tab. 1). In der Gruppe der Studierenden des neu strukturierten Seminars wurde die fachliche Kompetenz der Dozenten signifikant höher eingeschätzt (M_2016_ = 2,03, SD = 0,9 vs. M_2017_ = 1,6, SD = 0,67; *p* < 0,001) (Abb. 2a). Auch die Lernatmosphäre wurde durch das neue Kurskonzept verbessert (M_2016_ = 2,31; SD = 0,98 vs. M_2017_ = 1,94; SD = 1,0; *p* = 0,006) (Abb. 2b). Keine signifikanten Unterschiede zeigten sich für den Bezug zu aktuellen Themen sowie die Qualität der Unterrichtsmaterialien und der fächerübergreifenden Lehre (Tab. 1).

**Table 1:** 

	Mittelwert M_2016_ M_2017_	Standardabweichung	*p*-Wert
Wie bewerten Sie die Veranstaltung insgesamt?	2,56	1	0,011*
	2,23	0,91	
Wie gut wurden die angegebenen Lernziele definiert?	2,67	1,04	0,003*
	2,25	1,01	
Prüfungsrelevanz der Veranstaltung	2,28	1,01	0,007*
	1,93	0,83	
Wie schätzen Sie die fachliche Kompetenz des Dozenten ein?	2,03	0,9	< 0,001*
	1,6	0,67	
Wie bewerten Sie Motivation und Vorbereitung des Dozenten?	2,32	0,98	0,007*
	1,97	0,91	
Hat der Dozent die Inhalte angemessen wiederholt?	2,39	0,86	< 0,001*
	1,97	0,79	
Wie bewerten Sie die Möglichkeit, Fragen zu stellen, und die Bereitschaft zur Diskussion?	1,87	0,95	0,007*
	1,55	0,7	
Wie empfanden Sie die entstandene Lern- und Arbeitsatmosphäre?	2,31	0,98	0,006*
	1,94	1	
Konnten Sie dem Tempo des Dozenten gut folgen?	2,09	0,87	0,03*
	1,86	0,75	
Inwieweit wurden Bezüge zu aktuellen Themen hergestellt?	2,24	0,96	0,051
	2,01	0,83	
Wie bewerten Sie die Qualität der Unterrichtsmaterialien und -medien?	2,46	0,99	0,23
	2,35	0,99	
Wie gut wurde fächerübergreifend gelehrt?	2,5	0,96	0,071
	2,29	0,84	

*p* < 0,05, * zeigt signifikante Unterschiede zwischen den Gruppen

**Figure 3:**
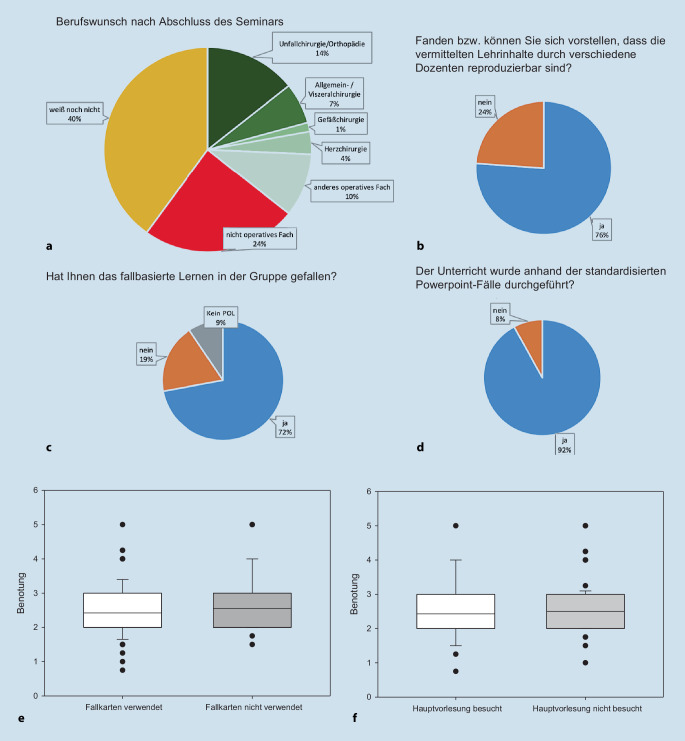

An der selbsterstellten Evaluation nahmen insgesamt 130 Studierende teil, die sich durchschnittlich im 6. Studiensemester befanden und das neu gestaltete Seminar absolviert hatten (41 % männlich zu 59 % weiblich). Der Berufswunsch der Studierenden nach Abschluss des Seminars zeigte, dass 24 % ein nichtoperatives Fach wählen wollten, 40 % es noch nicht wussten und 36 % eine Weiterbildung in einem operativen Fach machen wollten (Abb. 3a). Insgesamt fanden 76 % der Studierenden den Lerninhalt durch verschiedene Dozenten reproduzierbar (Abb. 3b). Dem Großteil der Studierenden gefiel das fallbasierte Lernen in der Gruppe (Abb. 3c). Das Praxisseminar wurde in 54 % der Fälle mithilfe der erstellten Fallkarten durchgeführt; in 92 % der Fälle wurden die erstellten Fallpräsentationen genutzt (Abb. 3d). Die Frage „Fanden Sie das Praxisseminar Unfallchirurgie gut strukturiert?“ wurde in 70 % mit ja beantwortet (30 % nein). Die Anforderungen des Praxisseminars wurden von 79 % der Studierenden als „genau richtig“ empfunden. 17 % waren unterfordert und 4 % überfordert. Die Themen bezüglich der klinischen Relevanz wurden überwiegend mit sehr gut (14 %) und gut (58 %) bewertet. 20 % empfanden die Relevanz befriedigend, 7 % ausreichend und 1 % mangelhaft. Durch das Praxisseminar konnten 54 % der Studierenden für das Fach Unfallchirurgie/Orthopädie motiviert werden. 23 % der Studierenden hatten vor dem Praxisseminar die Hauptvorlesung „Unfallchirurgie“ besucht. Weiter wurde auch im spezifischen zweiten Evaluationsbogen die neue Struktur in Schulnoten bewertet; hier ergab sich die Note 2,46 (SD = 0,79). Hierbei zeigte sich in der Subgruppenanalyse (Hauptvorlesung besucht vs. nicht besucht) kein Unterschied in der Benotung zwischen den Studierenden, die die Hauptvorlesung besucht hatten (2,43 SD = 0,93; *p* > 0,05), und der Bewertung der Gruppe der Studierenden, welche die Vorlesung nicht besucht hatten (2,5 SD = 0,735; *p* > 0,05) (Abb. 3e). Die Subgruppenanalyse zeigte außerdem: Studierende, welchen das fallbasierte Konzept gefiel, zeigten keinen Unterschied zwischen dem Einsatz von Fallkarten und ohne Fallkarten. Es zeigte sich auch kein Unterschied zwischen Studierenden, welche die Hauptvorlesung besucht hatten, und den Studierenden, welche die Hauptvorlesung nicht besucht hatten (Abb. 3f).

## Diskussion

Durch ein fallbasiertes Lehrkonzept für das unfallchirurgische Praxisseminar konnte dieses zu einer eher studierendenzentrierten Lehrveranstaltung verändert werden. Die schrittweise Analyse eines klinischen Falles und die Verknüpfung mit bereits vorhandenem Wissen sind dem klinischen Arbeitsalltag ähnlicher als klassische Lernmethoden wie z. B. Frontalunterricht. So wurden bereits verschiedene studierendenzentrierte Curricula entwickelt und publiziert [1, 2, 4, 10, 19, 25]. Qin et al. zeigten in einer Metaanalyse, dass diese Konzepte die Lehrumgebung der medizinischen Ausbildung verbessern können [17]. Witten-Herdecke berichtete als erste deutsche Universität über den Einsatz von problemorientiertem und einem damit fast vollständig studierendenzentrierten Lehrkonzept im medizinischen Bereich [9]. In der Literatur werden positive Erfahrungen und Chancen im Rahmen der Restrukturierung der Lehrveranstaltungen durch studierendenzentrierte Formate wie fallbasiertes Lernen oder POL beschrieben [6, 9, 17]. Ziel des POL ist es, die Lehrinhalte als Problem den Studierenden bewusst zu machen und für dieses eigenständig unter Zuhilfenahme von Literatur eine entsprechende Lösung zu finden. Dadurch ergeben sich neue Fragestellungen, welche dann tiefergehend bearbeitet werden können [9]. Eine alternative zu POL ist das fallbasierte Lernen. Durch die Moderation können Missverständnisse oder fehlerhafte Lösungsansätze frühzeitig vermieden werden. Dadurch kann die Lehrstruktur effizienter und zielgerichteter als im POL erfolgen [7, 20, 22]. Mithilfe des fallbasierten Lernens kann dadurch eine Balance zwischen dozenten- und studierendenzentrierter Lehre erreicht werden [7]. Diese kann, je nach Bedarf, dynamisch im Rahmen der Lehrveranstaltung verändert werden [7]. Dadurch kann fallbasiertes Lernen auch bei geringerem Vorwissen und Zeitlimitationen eingesetzt werden, während POL häufig ein umfassendes Eigenstudium mit Literaturrecherche und intensiver Vorbereitung benötigt [6].

Da die Hauptvorlesung Chirurgie im selben Semester stattfand, war das Vorwissen der Studierenden in Abhängigkeit vom Vorlesungsplan heterogen. Um die Einflussnahme des Dozenten möglichst zu reduzierten, ist das Vorwissen allerdings bei der klassischen Durchführung von problem- und fallbasierten Lehrkonzepten maßgeblich [12]. Da keine Veränderung des Gesamtcurriculums der Universität möglich war, musste das neue Lehrkonzept in das bereits bestehende Curriculum eingegliedert werden. Um dem Wissensunterschied vor dem Hintergrund der curricularen Rahmenbedingungen an unserer Fakultät zu begegnen, wurden zur Vorbereitung auf die Lehrveranstaltung Empfehlungen wie die Nutzung von eLearning-Angeboten über eine eigene Plattform [11, 12] und weiterführende Literatur gegeben. Im Seminar selbst wurden bedarfsgerecht Hilfestellungen gegeben und das standardisierte Lehrmaterial (Klassifikationshilfen, Therapieschritte etc.) entsprechend verwendet. Dadurch war stets ein positiver subjektiver Lernerfolg gewährleistet. Unsere Ergebnisse legen nahe, dass dadurch der Effekt nicht maßgeblich beeinflusst wurde, wie sich an der durchgeführten Subgruppenanalyse ohne Bewertungsunterschied in diesen Gruppen zeigt.

Der Berufswunsch nach dem Seminar zeigte, dass 40 % der Studierenden sich noch nicht sicher waren und 36 % der Studierenden eine Weiterbildung in einem chirurgischen Fach nach dem Studium anstreben. Im nationalen Vergleich entsprach das hier untersuchte Kollektiv anderen Umfragestudien von Studierenden [8]. 54 % der Studierenden konnten durch das Seminar für das Fach Orthopädie und Unfallchirurgie motiviert werden. Ob es sich hierbei um einen nachhaltigen Effekt handelt, kann anhand unserer Ergebnisse nicht gesichert werden. Allerdings ist eher von einem Einmaleffekt auszugehen, da nur 14 % der Studierenden eine Weiterbildung zum Facharzt Orthopädie/Unfallchirurgie nach dem Seminar anstrebten.

Die im neuen Curriculum verwendeten Fallbeispiele wurden anhand der DGUV Unfallstatistik 2016 so ausgewählt, dass 4 häufige Verletzungsmuster der jeweiligen Körperregion pro Seminartermin behandelt werden konnten. Dies entsprach den Inhaltsvorgaben des ursprünglichen Seminars. Im Rahmen des Seminars waren 20 Dozenten am Unterricht beteiligt. Durch diese erfolgte in 54 % der Einsatz der erstellten, papierbasierten Fallkarten, welche in Kleingruppen bearbeitet und anschließend mit der gesamten Seminargruppe weiter diskutiert wurden. Die elektronische Kasuistik wurde dagegen in 92 % der Fälle verwendet und in der Gruppe erarbeitet. Hierbei zeigte sich in der Bewertung des Seminars kein Unterschied zwischen den Gruppen, welche die papierbasierten Fallkarten verwendet hatten, gegenüber den Studierenden, welche die Kasuistik mittels elektronischer Präsentation besprochen hatten. Also konnte bereits durch die digitale Darstellung der Kasuistik und das gemeinsame Erarbeiten die unfallchirurgische Lehre verbessert werden. Dies zeigte sich auch in der Lernatmosphäre und der Diskussionsbereitschaft im Seminar. Beim fallbasierten Lehrformat zeigte sich eine Verbesserung, was im Einklang zu vorangegangenen Studien steht [5, 18]. Durch erhöhte Kommunikation im Seminar können dann auch Inhalte besser und gezielter wiederholt werden, wodurch auch Lernziele als besser definiert empfunden wurden. Diese Art der Kommunikation führte zu einer als gesteigert wahrgenommenen Motivation der Dozenten. Diese wurde rein subjektiv bewertet, allerdings kann durch Diskussionen im weitestgehend dozentenunabhängigen Lehrformat ein lebhaftes Seminar stattfinden. Dadurch kann auch die unterschiedliche Erfahrung der Dozierenden aus subjektiver Sicht der Studierenden ausgeglichen werden.

Der überwiegende Teil der Studierenden empfand das fallbasierte Konzept als eine sinnvolle Lehrmethode. Allerdings waren 19 % der Studierenden nicht von der Methode überzeugt. Dieser Effekt wurde auch in anderen, ähnlich konzipierten Studien beschrieben [18]. Dort zeigte sich keine Präferenz der Studierenden zu POL und somit studierendenzentrierter Methodik im Vergleich zur traditionellen Lehre [18]. In der Literatur wird die Ursache für die Ablehnung der Studierenden gegenüber studierendenzentrierten Lehrinhalten wie POL darin gesehen, das an den Universitäten meist dozentenzentriert gelehrt wird [13, 18]. Eine Umstrukturierung konnte in anderen Studien das positive Feedback für studierendenzentrierter Lehrmethoden deutlich erhöhen [15, 18, 24]. Eine weitere Möglichkeit, die Studierenden im Umgang mit eigenständigen, fallbasierten Lehrformaten zu fördern, aber auch Inhalte zu vertiefen, ist dabei, über die üblichen Seminare hinaus weitere Lehrinhalte und Kasuistiken über eLearning zur Verfügung zu stellen [11, 12].

Eine uneingeschränkte Wissensvermittlung durch fall- und problemorientiertes Lernen wird in der Literatur mitunter aber auch kritisch gesehen und ein reflektierter, gezielter Einsatz gefordert [5, 24]. Auch an die Dozenten werden durch diese Lehrmethoden höhere Anforderungen gestellt [10]. Das starke Miteinbeziehen der Studierenden, v. a. hinsichtlich der Fallvorstellung und Diskussion, erfordert sinnvoll ergänzende Informationen, um das Auftreten von Missverständnissen oder gar die Verbreitung von Fehlern während des Seminars zu verhindern [9]. In dezidiert dafür angelegten Seminaren können Fälle, wie die hier vorgestellten, aber ein gutes Instrument sein, um Studierende in die Lage zu versetzen, Verletzungsmuster zu analysieren und Behandlungskonzepte festzulegen [24]. Diese Lernmethode bereitet die Studierenden auf das klinische Arbeiten vor [14]. In der Folge ist es vorstellbar, dass die fallbasierten Methoden zusätzlich mit Training an chirurgischen Simulatoren kombiniert wird, um auch den praktischen Aspekt der Ausbildung abzubilden.

## Limitationen

Die Evaluationen dieser Studie erfolgte anhand der durch die Universität vorgegebenen Evaluationsbogen und anhand der spezifisch für die Neugestaltung erstellten Evaluation. Die spezifische Evaluation wurde nur in der Gruppe des neu etablierten Lehrkonzeptes durchgeführt, und nicht an der Kontrollgruppe aus dem Vorjahr. Die Dozenten setzten das neue Konzept um, allerdings waren die Fallkarten nicht in allen Seminaren verwendet worden, sodass dadurch Unterschiede in der Durchführung entstanden. Das elektronische Fallmaterial wurde dabei aber von über 90 % der Dozierenden entsprechend eingesetzt. Hinsichtlich der Bewertungsqualität zwischen den papierbasierten und elektronischen Unterrichtsabläufen gab es in der Subgruppenanalyse allerdings keine Unterschiede, sodass davon auszugehen ist, dass ein rein elektronisches, fallbasiertes Kursformat ausreichend ist.

## Ausblick

Die hier durchgeführte Untersuchung zeigte, dass durch fallbasiertes Lernen die Motivation der Studierenden erhöht werden kann. Durch das studierendenzentrierte Lehrkonzept können auch junge und somit weniger erfahrene Dozenten kompetenter wahrgenommen werden. Die Einführung solcher Lehrkonzepte ermöglicht es Studierenden, sich mit Erkrankungen und den Fällen intensiver zu beschäftigen. Diese Angebote können dann schrittweise z. B. zusätzlich in eLearning-Angebote eingebunden werden. Des Weiteren können die Fälle weiterentwickelt werden und später zu noch studentenzentrierterem problemorientiertem Lernen verwendet werden. Somit kann die Rolle des Dozenten schrittweise reduziert werden. Dies ermöglicht es, Studierende durch eigenständige Problemlösung auf die spätere klinische Tätigkeit vorzubereiten. Eine noch aus unserer Sicht unbeantwortete Frage ist, ob Seminarzeiten durch studierendenzentrierte Lehre effizienter genutzt werden können. Ein Großteil des vermittelten Lehrinhalts könnte dann bereits im Rahmen der Vorbereitung erarbeitet werden und dann im Seminar durch die Anwendung des Wissens vertieft werden.

### Infobox 1 Zusammenstellung der Kasuistikthemen

*Obere Extremität und Thorax*

Schulterluxation

Proximale Humerusfraktur – einfacher Frakturtyp

Proximale Humerusfraktur – mehrfragmentärer Frakturtyp

Pneumothorax

*Untere Extremität*

Tibiakopffraktur

Mediale Schenkelhalsfraktur

Pertrochantäre Femurfraktur

Unterschenkelfraktur

*Hand und Handgelenk*

Distale Radiusfraktur

Distale Radiusfraktur des Kindes

Mittelhandfraktur

Skaphoidfraktur

*Wirbelsäule und Becken*

Densfrakturen

Fraktur der LWS/BWS

Beckenfraktur

Beckenfraktur mit kombinierter Acetabulumfraktur

*Fuß und Sprunggelenk*

Sprunggelenkluxationsfraktur

Kalkaneusfraktur

Lisfranc-Luxation

Talusfraktur

### Infobox 2 Evaluationsbogen der Universität zum semesterübergreifenden Vergleich

*Evaluationsbogen 1*

Wie bewerten Sie die Veranstaltung insgesamt?

Inwieweit wurden Bezüge zu aktuellen Themen hergestellt?

Inwieweit wurden medizinische/zahnmedizinische/klinische Bezüge hergestellt?

Wie gut wurden die angegebenen Lernziele definiert?

Inwieweit schätzen Sie die Veranstaltung prüfungsrelevant ein?

Wie bewerten Sie die Qualität der Unterrichtsmaterialien und -medien?

Wie schätzen Sie die fachliche Kompetenz des/der Dozenten ein?

Wie bewerten Sie die Motivation und Vorbereitung des/der Dozenten?

Konnten Sie dem Tempo des Dozenten gut folgen?

Hat der Dozent die Inhalte angemessen wiederholt?

Wie bewerten Sie die Möglichkeit, Fragen zu stellen, und die Bereitschaft zur Diskussion?

Wie gut wurde fächerübergreifend gelehrt?

Wie empfanden Sie die entstandene Lern- und Arbeitsatmosphäre?

### Infobox 3 Für das Projekt entwickelter, zusätzlicher Evaluationsbogen

*Evaluationsbogen 2*

Welche Erwartungen hatten Sie an das Praxisseminar Unfallchirurgie?

Was ist Ihr Berufsziel?

Welche Erwartungen hätten Sie an den Kurs, wenn Sie ihn veranstalten würden?

Semester

Geschlecht

Alter

Wie schätzen Sie Ihre handwerkliche Geschicklichkeit ein?

Wie fanden Sie die gewählten Themen für den Kurs bezüglich ihrer klinischen Relevanz?

Wie empfanden Sie die Anforderungen des Praxisseminars?

Hat Ihnen das fallbasierte Lernen in der Gruppe gefallen?

Fanden Sie das Praxisseminar Unfallchirurgie gut strukturiert?

Fanden Sie das Praxisseminar Unfallchirurgie unabhängig vom durchführenden Dozenten standardisiert?

Fanden bzw. können Sie sich vorstellen, dass die vermittelten Lehrinhalte durch unterschiedliche Dozenten reproduzierbar sind?

Wurde das Praxisseminar mit den erstellten Lernkarten (Din-A4-Umschlägen mit Gruppenarbeit) durchgeführt?

Der Unterricht wurde anhand der standardisierten PowerPoint-Fälle durchgeführt?

- *Fanden Sie die entstandene Lernatmosphäre gut?*
- *Hätten Sie sich mehr aktive Mitarbeit gewünscht?*
- *Wenn Ja, wie?*
- *Konnten Sie den Lerninhalten folgen?*

Hat Sie der Kurs animiert, sich für das Fach Orthopädie/Unfallchirurgie zu begeistern?

Hatten Sie vor dem Kurs bereits die Vorlesung „Unfallchirurgie“ besucht?

Halten Sie es für sinnvoller, das Praxisseminar in einem höheren Semester (nach dem Abschluss der Hauptvorlesung Chirurgie) ins Curriculum einzubinden?

Welche Schulnote würden Sie dem neu strukturierten Praxisseminar Unfallchirurgie geben?

Haben Sie Verbesserungsvorschläge? Wenn ja, welche:

## Fazit für die Praxis

Durch das neu eingeführte fallbasierte Lehrkonzept im Praxisseminar Unfallchirurgie konnte eine studierendenzentrierte Lehre etabliert werden. Hierbei zeigte sich, dass das Konzept durch die Studierenden überwiegend positiv, insbesondere hinsichtlich der klinischen Relevanz und Lernatmosphäre, empfunden wurde und die wahrgenommene Motivation der Dozenten für das Seminar gesteigert werden konnte. Eine rein elektronische Fallvorstellung scheint dabei ausreichend. Standardisierte fallbasierte Unterrichtskonzepte in der Unfallchirurgie stellen in unseren Augen eine sinnvolle Ergänzung zu bestehenden Lehrmethoden dar.
